# A case report of visceral leishmaniasis and malaria co-infection with pancytopenia and splenomegaly - a diagnostic challenge

**DOI:** 10.1186/s12879-019-4478-1

**Published:** 2019-10-15

**Authors:** Pragya Gautam Ghimire, Prasanna Ghimire, Jyoti Adhikari, Anurag Chapagain

**Affiliations:** 1 Department of Pathology, Nepalgunj Medical College and Teaching Hospital, Kohalpur, Banke 21904 Nepal; 2 Department of Radiology, Nepalgunj Medical College and Teaching Hospital, Banke, 21904 Banke Nepal; 3 Department of Pediatrics, Nepalgunj Medical College and Teaching Hospital, Banke, 21904 Banke Nepal; 4 Department of Emergency, Bheri Provincial Hospital, Banke, Banke Nepal

**Keywords:** Leishmaniasis, Malaria, Co-infection, P.vivax

## Abstract

**Background:**

Leishmaniasis and malaria are tropical diseases with more than half of the world population at risk of infection resulting in significant morbidity and mortality. Co-infection of Leishmaniasis and malaria pose a great challenge in the diagnosis as well as overall management.

**Case presentation:**

In this case report, we present a rare case of a 5 years old child hailing from non-endemic region of Nepal with history of fever for a period of 3 months who was diagnosed as co-infection of malaria due to *Plasmodium vivax* and visceral Leishmaniasis with pancytopenia that subsequently improved after a course of treatment.

**Conclusions:**

A high index of suspicion for a possibility of co-infection with Leishmaniasis and malaria should be borne in mind when an individual hailing from or having history of travel to endemic countries presents with prolonged fever.

## Background

Leishmaniasis and malaria are tropical diseases with endemecity noted in various regions of the world. Both the diseases are of public health problems resulting in significant morbidity and mortality even with treatment. An early diagnosis is important in management of cases [1]. Although few studies have demonstrated co – infection of visceral Leishmaniasis and malaria in certain African and Middle East populations, such association noted in South Asia is limited [2]. We present a case of visceral Leishmaniasis and Malaria co-infection complicated with pancytopenia and fever in a child hailing from Jajarkot District of Karnali Region, a non-endemic region of Malaria posing a diagnostic challenge. To our knowledge, no such cases have been reported from Nepal.

## Case presentation

**Fig. 1:**
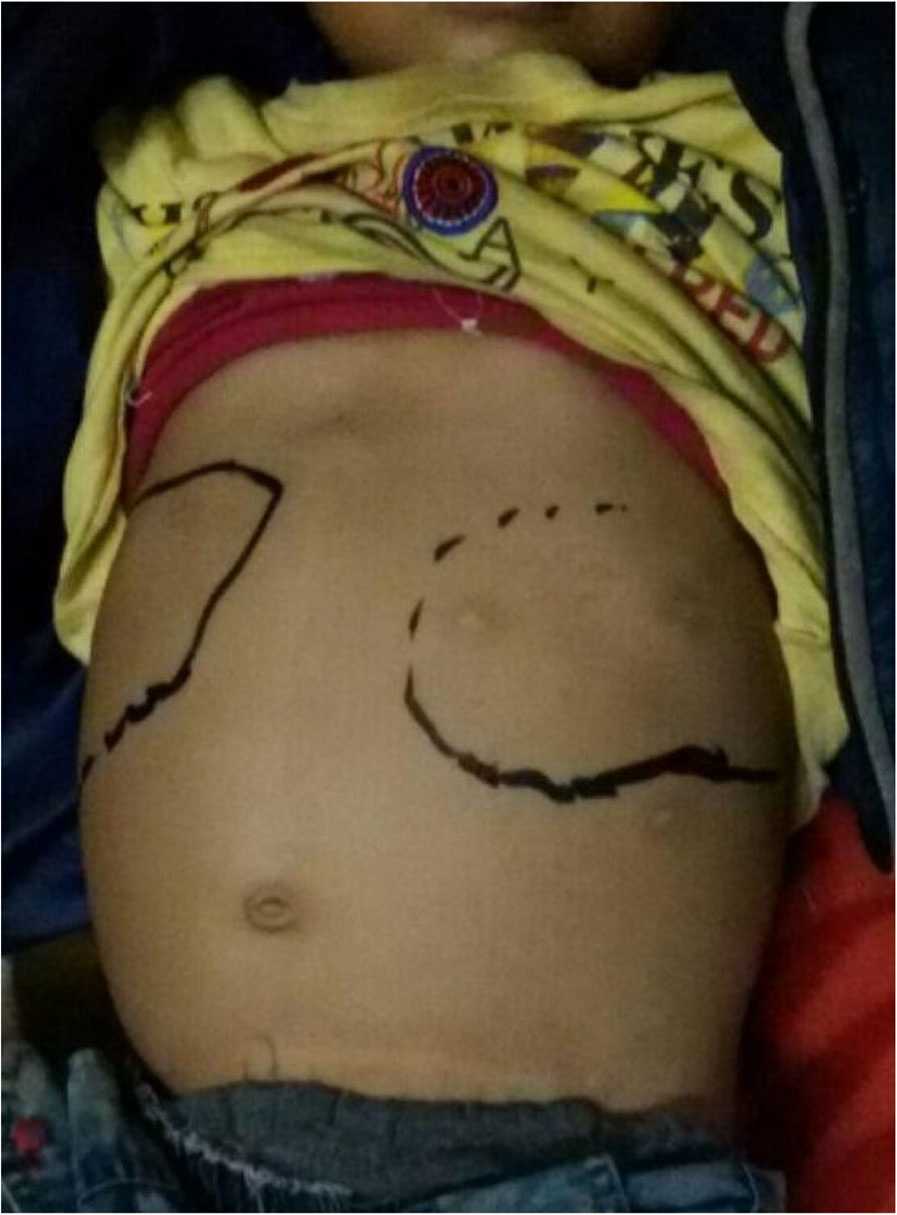
Photograph of the patient demonstrating hepatosplenomegaly

**Fig. 2:**
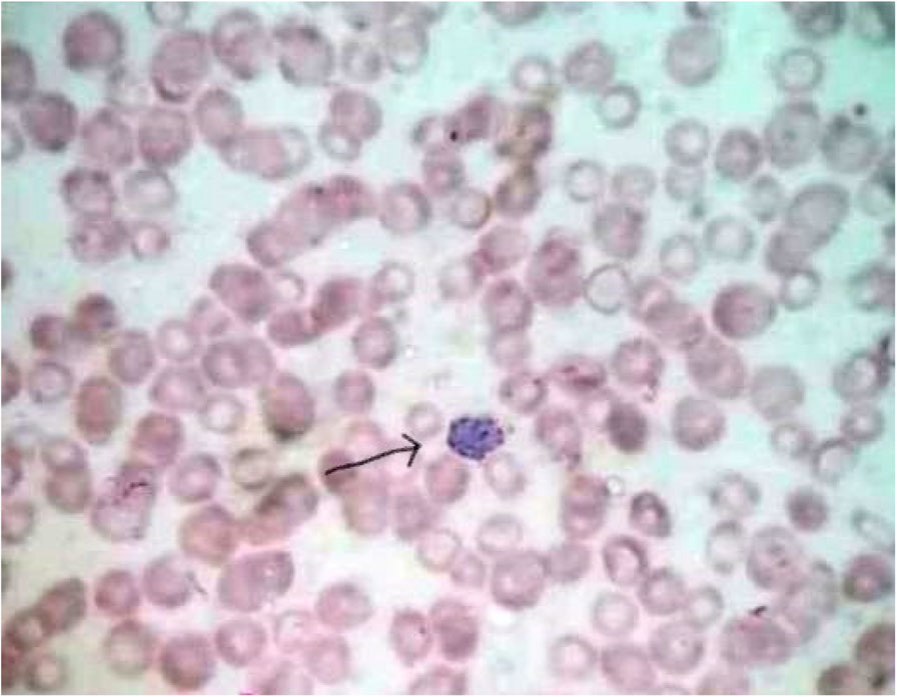
Photomicrograph showing schizont of *P. vivax* malaria in thicker portion of the film. (Wright stain, X 100)

**Fig. 3:**
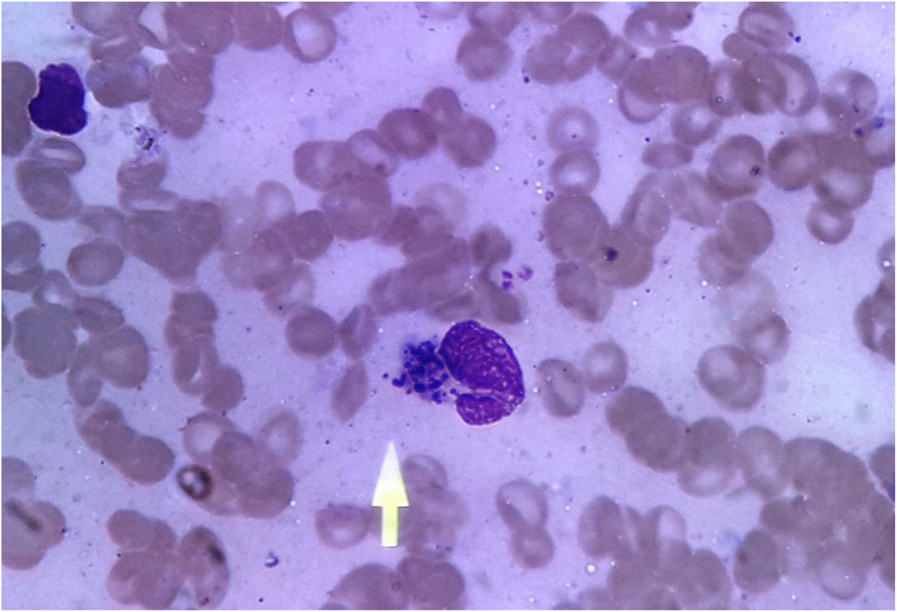
Leishmania Donovani (LD bodies inside a monocyte. (Wright stain, X 100)

A-5 year- old child hailing from a rural hilly region of Nepal presented with history of on and off high grade fever associated with chills and rigor, abdominal pain and constipation for a duration of 3 months. Patient was treated locally for his symptoms that did not resolve which was then referred to our hospital for further management. On physical examination, patient was febrile, hepatosplenomegaly was noted (Fig. 1). An ultrasound examination confirmed hepatosplenomegaly. Laboratory investigation demonstrated pancytopenia with hemoglobin – 7.2 g/dl, RBC 2.94 × 10^12/^L, WBC 2.90 × 10^9^/L, Platelet count- 63 × 10^9^/L. On peripheral blood smear, schizont of *P. vivax* malaria as well as intracellular Leishmania-Donovan (LD) bodies were noted (Figs. 2 and 3). rK 39 immunochromatographic and malaria rapid diagnostic test (RDT) for *P. vivax* were positive. Child was treated with chloroquine phosphate with first and second dose of 10 mg base/ kg/ at 24 h interval followed by third dose of 5 mg base/ kg after 24 h. Child was referred to nearby government hospital for a free supply of single dose of liposomal amphotericin B (10 mg/kg) as infusion. Child was also started on Primaquine phosphate (0.5 mg/kg/day) for 15 days. Patient had significantly improved clinically and a blood film on follow up demonstrated parasite clearance.

## Discussion and conclusions

Malaria as a tropical disease is of significant public health problem worldwide with an estimated 3.2 billion people at risk and with ongoing malaria transmission in 91 countries. In Nepal, southern plains which has border with India are endemic for malaria with majority of the cases due to *Plasmodium vivax* [3]. Although, there has been a significant improvement towards achieving malaria elimination in Nepal, still significant number of cases is annually recognized. A major problem which is noted is the significant co-infection of malaria with other diseases that has not been addressed and programmes not tailored to curtail such cases [4].

Another tropical disease of much concern in South Asia with significant morbidity and mortality is Leishmaniasis. The southern belt (Terai region) of Nepal is also endemic for visceral Leishmaniasis. Major control programmes are concentrated in these regions. However, there has been a geographical variation in cases of Leishmaniasis recently. Few cases are even noted in Hilly regions of Nepal [1]. Moreover, studies have demonstrated that cutaneous Leishmaniasis is an under recognized presentation of Leishmaniasis with significant cases noted in individuals inhabiting in the Terai belt as well as hilly region [5, 6].

Malaria and visceral Leishmaniasis co-infection are noted in certain African studies constituting almost 4.1% in endemic regions. There is dearth of literature of cases of co-infection in Nepal [2]. Certain studies have demonstrated co-infection prevalence rate ranging from 3.8 to 60.8% [7]. Our case was a child who was treated locally for on and off fever and demonstrated co-infection for *P. vivax* and Leishmaniasis. In our case, splenomegaly was present with no evidence of infarction on ultrasound. Few cases of co-infection have been reported with splenic infarction [8]. Our case is unique with presence of pancytopenia which although sometimes noted with *P. falciparum* infection is a rare presentation of *P. vivax* infection. Moreover, treatment with chloroquine is often not effective requiring other drugs including Artesunate [9]. Since, our patient hailed from choloroquine sensitive areas, oral chloroquine was used a first line antimalarial drug along with injectable liposomal amphotericin B.

Our case has highlighted potential areas of concern in endemic regions for malaria and Leishmaniasis. Firstly, in a resource limited settings as ours, vector control programs should be in an integrated and coordinated manner addressing endemic diseases as a whole and not indivualised; high risk areas and population groups should be identified beforehand. Routine screening for possible co-infection should be performed as delay in diagnosis and treatment results in significant morbidity and mortality. Besides, potential treatment failure and adverse reactions while managing such cases should be understood and planned accordingly. A high index of suspicion for a possibility of a co-infection is to be made when individuals hailing from endemic regions present with fever.

## Data Availability

The datasets used and/or analyzed during the current study are available from the corresponding author on reasonable request.

## Abbreviations

LD: Leishmania donovani
*P. vivax*: *Plasmodium vivax*
RBC: Red blood cell count
rK39: Ribosomal kininogen
WBC: White blood cell count
